# The Efficacy and Safety of Etrasimod for the Induction and Maintenance of Remission in Moderate to Severe Ulcerative Colitis: An Updated Systematic Review and Meta-Analysis of Randomised Controlled Trials

**DOI:** 10.7759/cureus.116966

**Published:** 2026-09-26

**Authors:** Chaudhry Zaid Riaz, Muhammad Raza, Iman Afraz, Eymaan Riaz Chaudhry, Aima Aslam, Aira Anwaar, Ali Jamshed Qureshi, Misha Jannat, Abdul Raffeh Basit, Muhammad Waleed Ahmed, Muhammad Hassan Qureshi

**Affiliations:** 1 Internal Medicine, NHS England Education East Midlands, Nottingham, GBR; 2 Medicine, Combined Military Hospital (CMH) Lahore Medical College and Institute of Dentistry, Lahore, PAK; 3 Internal Medicine, Combined Military Hospital, Lahore, PAK; 4 Internal Medicine, Allegheny General Hospital, Allegheny Health Network, Pittsburgh, USA; 5 Medicine, Jinnah Sindh Medical University, Karachi, PAK; 6 Medicine, Lahore Medical and Dental College, Lahore, PAK; 7 Medicine, Ameer-ud-Din Medical College, Lahore, PAK; 8 Internal Medicine, UCHealth Parkview Hospital, Pueblo, USA; 9 Public Policy and Health, Queen Mary University of London, London, GBR; 10 Emergency Medicine, University Hospital Kerry, Tralee, IRL; 11 Emergency Medicine, Combined Military Hospital, Lahore, PAK; 12 Public Health and Policy, Anglia Ruskin University, Cambridge, GBR

**Keywords:** etrasimod, inflammatory bowel diseaes (ibd), sphingosine 1-phosphate, sphingosine-1-phosphate receptor modulator, ulcerative colıtıs

## Abstract

Etrasimod is a selective sphingosine 1-phosphate (S1P) receptor modulator that is used as a novel oral treatment for moderate to severe ulcerative colitis (UC). The efficacy and safety of Etrasimod across both induction and maintenance treatment phases require further evaluation. An extensive search was conducted across PubMed, ClinicalTrials.gov and Cochrane Central Register of Controlled Trials (CENTRAL), identifying seven reports of five independent randomised controlled trials (RCTs) comprising 1230 participants across multiple countries to be included in this meta-analysis. Data analysis was carried out using a fixed-effects model, with results reported as risk ratios (RRs) and 95% confidence intervals (CIs). Etrasimod significantly increased the likelihood of clinical remission at Week 12 (RR 2.63; 95% CI: 1.91, 3.62; p-value < 0.00001) and beyond 40 weeks (RR 2.87; 95% CI: 1.89, 4.37; p-value < 0.00001). Clinical response also improved significantly at Week 12 (RR 1.68; 95% CI: 1.44, 1.95; p-value < 0.00001) and beyond 40 weeks (RR 1.73; 95% CI: 1.35, 2.21; p-value < 0.0001). Etrasimod significantly improved endoscopic outcomes at Week 12 (RR 2.15; 95% CI: 1.67, 2.75; p-value < 0.00001) and beyond 40 weeks (RR 2.57; 95% CI: 1.79, 3.68; p-value < 0.00001). Significant improvements were also observed in symptomatic remission at Week 12 (RR 1.81; 95% CI: 1.47, 2.24; p-value < 0.00001) and beyond 40 weeks (RR 1.96; 95% CI: 1.47, 2.61; p-value < 0.00001), as well as mucosal healing at Week 12 (RR 2.67; 95% CI: 1.77, 4.02; p-value < 0.00001) and beyond 40 weeks (RR 2.62; 95% CI: 1.69, 4.04; p-value < 0.0001). Steroid-free clinical remission was also significantly more likely with Etrasimod (RR 3.83; 95% CI: 2.21, 6.62; p-value < 0.00001). No statistically significant difference was observed in serious adverse events between Etrasimod and placebo (RR 1.06; 95% CI: 0.61, 1.85; p-value = 0.83), although a small increase in other adverse events was observed with Etrasimod (RR 1.15; 95% CI: 1.03, 1.28; p-value = 0.01). No deaths were reported in either treatment group. The findings of this meta-analysis support Etrasimod as an effective and well-tolerated treatment option for achieving and maintaining remission in patients with moderate to severe UC. Further research will help to better understand the long-term effects and safety of Etrasimod in patients with different disease characteristics, comorbidities and potential drug interactions.

## Introduction and background

Ulcerative colitis (UC) is a lifelong disease that causes chronic gastrointestinal inflammation and ulcers. UC is further classified based on the region of involvement; ulcerative proctitis affects the rectum, left-sided colitis affects the left side of the colon and pancolitis involves the entire colon [1]. It affects the superficial gut lining of the large intestine [2]. UC is estimated to affect as many as 1.5 million people in the U.S. and approximately five million individuals worldwide, most commonly affecting individuals aged 15 to 30 [1,3,4]. This lifelong condition can be managed but cannot be cured [5]. The disease manifests with symptoms ranging from abdominal pain, chronic diarrhoea (which may be bloody), unintended weight loss, loss of appetite and fatigue [1,6]. The symptoms of UC vary in intensity from person to person and may change over time [5]. Individuals with UC experience disease flare-ups alternating with periods of remission [1]. Long-term complications include an increased risk of colon cancer, perforated bowel, and toxic megacolon [1,6]. UC is not limited to the GI tract; some patients experience extra-intestinal complications including osteoporosis, primary sclerosing cholangitis, swollen joints, eye pain and irritation and anaemia [1,6]. 

The exact cause of UC is unknown, but multiple factors likely play a role in its development. It is caused by an abnormal immune response in which the immune system attacks cells in the digestive tract [1,6]. There is a clear role of heredity, with family history and certain genetic mutations associated with increased risk of developing UC [5]. Environmental triggers are also believed to play a role. There is a range of risk factors associated with UC, including but not limited to age, ethnicity (increased risk in Ashkenazi Jews), family history, stress and antibiotics [1,6]. UC is not triggered by certain dietary habits, but certain foods like caffeinated drinks, greasy food, dairy products, alcohol and carbonated drinks may aggravate the symptoms [1,5,6,7]. 

UC management focuses on managing the immune response and inflammation. There is no standard treatment regimen; it is tailored to the individual based on their disease sub-type, severity and symptoms. The goals of management are to first achieve a rapid clinical improvement with normal biomarkers and second, to sustain disease remission and attain endoscopic normalisation in order to prevent long-term sequelae [3]. Medical management revolves around reducing the inflammatory response with medications, including aminosalicylates, corticosteroids, immunomodulators like 6-mercaptopurine (6-MP), methotrexate and biologic therapies like adalimumab, golimumab and infliximab [1,5,6]. Individuals may also benefit from antidiarrheal agents and antibiotics [1,6]. Although medical management strategies are expanding, approximately 30% of individuals require proctocolectomy at some point [1].

Etrasimod is a synthetic selective sphingosine 1-phosphate (S1P) receptor modulator that selectively activates S1P receptor subtypes 1, 4 and 5. Etrasimod is an advanced treatment option for patients with moderate to severe UC who require escalation of treatment or in patients where advanced therapy is being considered. Etrasimod is different from earlier non-selective S1P receptor modulators because it selectively acts on S1P receptor subtypes mentioned earlier. It may be used as monotherapy or alongside other appropriate background treatment for UC. S1P receptors are membrane-associated lysophospholipid signalling molecules that play a role in lymphocyte trafficking. The manner in which Etrasimod exerts its therapeutic effect in UC involves the reduction of lymphocyte migration into the intestines [8-10]. Etrasimod partially and reversibly inhibits lymphocytes from exiting the lymphoid tissues, leading to a decreased number of lymphocytes in the peripheral blood, thereby exerting its immunosuppressive effect. Etrasimod is a once-daily oral immunomodulator drug that is FDA-approved for the treatment of moderately to severely active UC [11].

Etrasimod has been studied for both the induction and maintenance of remission in patients with UC. Early studies evaluated the efficacy and safety of Etrasimod during the induction period, while later trials provided further evidence for its use during both induction and long-term treatment. Additional studies have provided data on outcomes beyond 40 weeks. Together, these studies provide evidence on efficacy and safety outcomes. The available evidence therefore allows assessment of both the short-term efficacy of Etrasimod at 12 weeks and its long-term effects during maintenance treatment.

This updated meta-analysis was designed to assess the effectiveness and safety profile of Etrasimod for induction and maintenance of remission in patients with moderate to severe UC. Previous meta-analyses have been limited by a smaller number of randomised controlled trials (RCTs) and did not provide the same assessment of long-term maintenance outcomes. In contrast, our meta-analysis includes seven reports of five independent RCTs incorporating more recent studies. We assessed a broad range of efficacy and safety outcomes during both induction at 12 weeks and maintenance beyond 40 weeks. This provides a more complete and up-to-date assessment of the clinical benefits and safety profile of Etrasimod. Given the increasing use of Etrasimod, updated evidence is needed on its effectiveness and safety profile. Our study aims to assess its effectiveness in achieving and maintaining remission and provide updated evidence for healthcare professionals and researchers.

## Review

Methods

This meta-analysis was designed and conducted in accordance with Preferred Reporting Items for Systematic Reviews and Meta-Analyses (PRISMA) guidelines [12]. The ability of the drug Etrasimod to improve the efficacy and safety outcomes for the induction and maintenance of remission in adult patients with moderate to severe UC is the research question which forms the basis of this meta-analysis.

Search Strategy

PubMed, ClinicalTrials.gov and Cochrane Central Register of Controlled Trials (CENTRAL) were used to identify relevant studies for this meta-analysis. The keywords used for database search included ‘Etrasimod’, ‘APD334’, ‘Ulcerative Colitis’, ‘UC’, ‘inflammatory bowel disease’ and ‘IBD’ using ‘OR’ and ‘AND’ as Boolean operators. Search terms related to RCTs, including ‘RCT’, ‘trial’ and ‘randomised controlled trial’, were also used in databases where appropriate filters for study design could not be applied. The literature search was last conducted in April 2026. The list of relevant studies was later screened manually. The inclusion and exclusion criteria for this meta-analysis are detailed below in Table 1.

**Table 1:** Inclusion and exclusion criteria

	Inclusion Criteria	Exclusion Criteria
Study Design	Only randomised controlled trials (RCTs) were included.	Reviews, case reports, observational studies, editorials, letters and pre-clinical or experimental studies were excluded.
Population	Adult patients with pre-existing moderate to severely active ulcerative colitis.	Paediatric population, mild or undiagnosed ulcerative colitis.
Intervention	Studies in which Etrasimod was administered.	Studies that combined Etrasimod with any drug not relevant to standard ulcerative colitis treatment.
Comparator	Placebo or no treatment.	Studies without a control group.
Study Outcomes	Studies reporting clinical remission, clinical response, endoscopic improvement, symptomatic remission, mucosal healing, steroid-free clinical remission and safety outcomes.	Studies that did not report any of the pre-specified efficacy or safety outcomes of interest.
Trial Identification	Reports from eligible RCTs were included, and multiple reports from the same trial were treated as one trial to avoid duplication.	Duplicate reports, duplicate trial records and reports that did not provide additional relevant information from an eligible RCT were excluded.
Language	Studies reported in English.	Studies in any other language besides English.

Data Extraction

Microsoft Excel (Microsoft® Corp., Redmond, WA) was used to retrieve and record data extracted from included RCTs. Two reviewers were used to carry out data extraction independently, eliminating any risk of bias. 

Ethical Approval and Quality Assessment

Quality assessment was carried out by two reviewers independently using the ‘Risk of Bias’ tool for randomised controlled trials (RoB 2.0) tool for randomised trials [13]. This study involved no first-hand collection of patient data and only used published data from previous RCTs; hence, no ethical approval was required.

Statistical Analysis

RevMan version 5.4 (The Cochrane Collaboration, London, UK) was used to perform statistical analysis. Risk ratio (RR) was used for analysis of study outcomes, and a 95% confidence interval (CI) was reported in association with the findings. I2 value was used to report heterogeneity. A fixed effects model was used for analysis, as RCTs were mostly similar.

Results

Study Selection

EndNote Online Classic (Clarivate, Philadelphia, PA) was used to screen the search results from all databases for relevance and removal of duplicate records. Figure 1 illustrates the study selection process in the PRISMA diagram. A total of 191 records were identified through database searches, including 21 from PubMed, seven from ClinicalTrials.gov and 163 from CENTRAL. After removing 63 duplicate records, 128 records were screened, which were assessed for relevance independently by two reviewers based on their titles and abstracts. A total of 36 records were excluded during this stage, including 27 based on their titles and nine after going through their abstracts. The remaining 92 records were considered for further assessment. Seventy-five records were removed in order to avoid duplication, as they were reports, analyses, registry records or other publications related to the same trials. The remaining 17 records were selected for retrieval, of which three could not be accessed or did not provide relevant data. Seven articles were excluded after full-text assessment because they did not meet the inclusion criteria, including registry records, studies in healthy volunteers and studies with designs other than RCTs. Finally, seven reports met the eligibility criteria and were included in the meta-analysis. 

**Figure 1:**
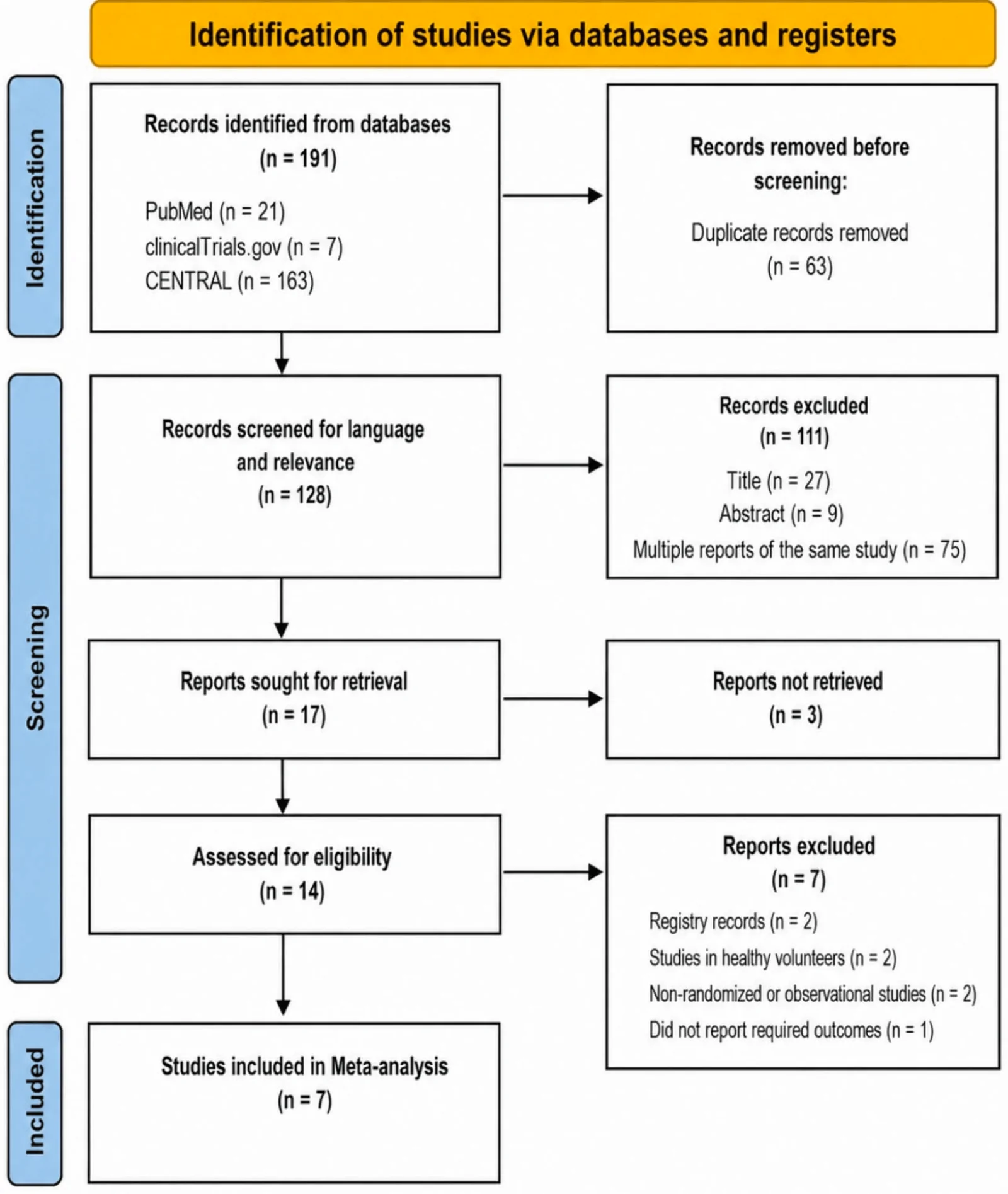
PRISMA diagram Reference: [12]

Study Characteristics

Seven reports representing five independent RCTs were included in our meta-analysis [14-20]. All included studies were randomised, double-blind, placebo-controlled trials. Two of the included reports were extension studies of previously identified parent trials. NCT02536404 was a 34-week extension of NCT02447302, and NCT04706793 was a 40-week extension study involving Japanese participants from NCT03996369 [14,15,17,20]. These reports were considered follow-up studies of their respective parent trials and were not treated as independent RCTs. Participants from the extension reports were not considered as additional participants when determining the number of independent trials or the total study population. Extension data were only used for relevant long-term outcomes. The pooled sample size was 1230 participants, with mean ages ranging from 40.4 to 44.0 years. Male participants accounted for approximately 55-65% of the study population. The included trials were conducted across multiple countries with treatment durations ranging from 12 to 52 weeks. The studies assessed a range of efficacy and safety outcomes, including clinical remission, clinical response and endoscopic improvement as the primary efficacy outcomes. Secondary outcomes included symptomatic remission, mucosal healing and steroid-free clinical remission, while safety outcomes included serious and other adverse events and all-cause mortality. Outcomes were assessed during both induction at 12 weeks and maintenance beyond 40 weeks. Study characteristics of the included RCTs are given in Table 2.

**Table 2:** Characteristics of included studies [14-20]

Study	Year	Country	Sample Size	Male, n (%)	Age	Treatment Duration	Study Design/Phase	Main Outcomes
NCT02447302 (OASIS) [14]	2021	21 countries	156	89 (57.1%)	42 ± 13.25	12 weeks	Phase 2, randomised, double-blind, placebo-controlled trial	Clinical remission, clinical response, endoscopic improvement, safety outcomes
NCT02536404 (OASIS OLE) [15]	2021	21 countries	118	71 (60.2%)	44.0 ± 13.30	Additional 34 weeks; 52 weeks total including parent study	34-week extension of NCT02447302	Clinical remission, clinical response, safety outcomes
NCT03945188 (Elevate UC 52) [16]	2022	40 countries	433	240 (55.4%)	40.4 ± 14.02	52 weeks	Phase 3, randomised, double-blind, placebo-controlled trial	Clinical remission, clinical response, endoscopic improvement, symptomatic remission, mucosal healing, steroid-free clinical response, safety outcomes
NCT03996369 (Elevate UC 12) [17]	2022	41 countries	354	208 (58.8%)	40.4 ± 13.40	12 weeks	Phase 3, randomised, double-blind, placebo-controlled trial	Clinical remission, clinical response, endoscopic improvement, symptomatic remission, mucosal healing, safety outcomes
NCT04607837 (Gladiator UC) [18]	2025	18 countries	233	130 (55.8%)	41.3 ± 13.08	52 weeks	Phase 2, randomised, double-blind, placebo-controlled trial	Clinical remission, clinical response, endoscopic improvement, symptomatic remission, mucosal healing, steroid-free clinical remission, safety outcomes
NCT05061446 (Japan Phase 2) [19]	2024	Japan	54	35 (64.8%)	43.3 ± 12.45	12 weeks + 4-week follow-up	Phase 2, randomised, double-blind, placebo-controlled trial	Clinical remission, clinical response, endoscopic improvement, symptomatic remission, mucosal healing, safety outcomes
NCT04706793 (Elevate UC 40 Japan) [20]	2024	Japan	42	25 (59.5%)	44.0 ± 14.06	40-week extension; 52 weeks total including parent study	40-week extension of Japanese participants from NCT03996369	Clinical remission, endoscopic improvement, symptomatic remission, mucosal healing, steroid-free clinical remission, safety outcomes

Risk of Bias Assessment

The RoB 2.0 was used for risk of bias assessment of the five independent RCTs included in this meta-analysis [13]. The seven included reports corresponded to these five RCTs; therefore, extension reports were not assessed separately for risk of bias assessment. Two reviewers independently performed the assessment, and any discrepancies were resolved by a third reviewer. The assessment covered five domains, including the randomisation process, deviations from intended interventions, missing outcome data, measurement of outcomes and selection of the reported results. Overall, the included reports were considered high-quality studies. Figure 2 shows the results of bias assessment of the five included RCTs.

**Figure 2:**
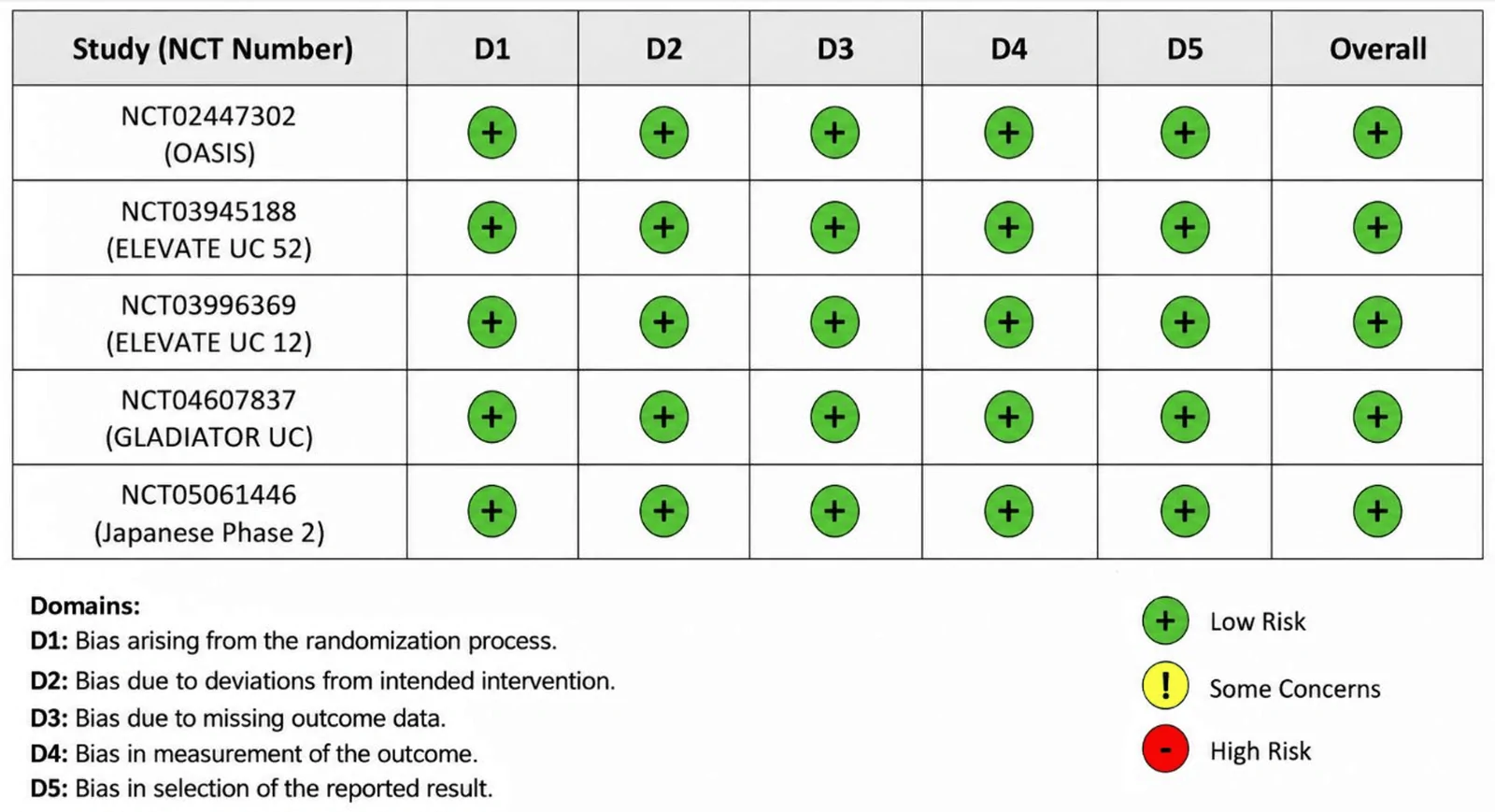
Risk of bias assessment References: [14,16-19]

Synthesis of Results

During the induction phase, the efficacy of Etrasimod was assessed at 12 weeks across the included studies.

Clinical remission: A statistically significant difference was observed in patients receiving Etrasimod compared with placebo in achieving clinical remission at Week 12 (RR 2.63; 95% CI: 1.91, 3.62; p-value < 0.00001). Patients receiving Etrasimod were 2.63 times more likely to achieve clinical remission at Week 12 in comparison to those receiving placebo. Heterogeneity was moderate (I² = 38%). Figure 3 illustrates the forest plot of these findings.

**Figure 3:**
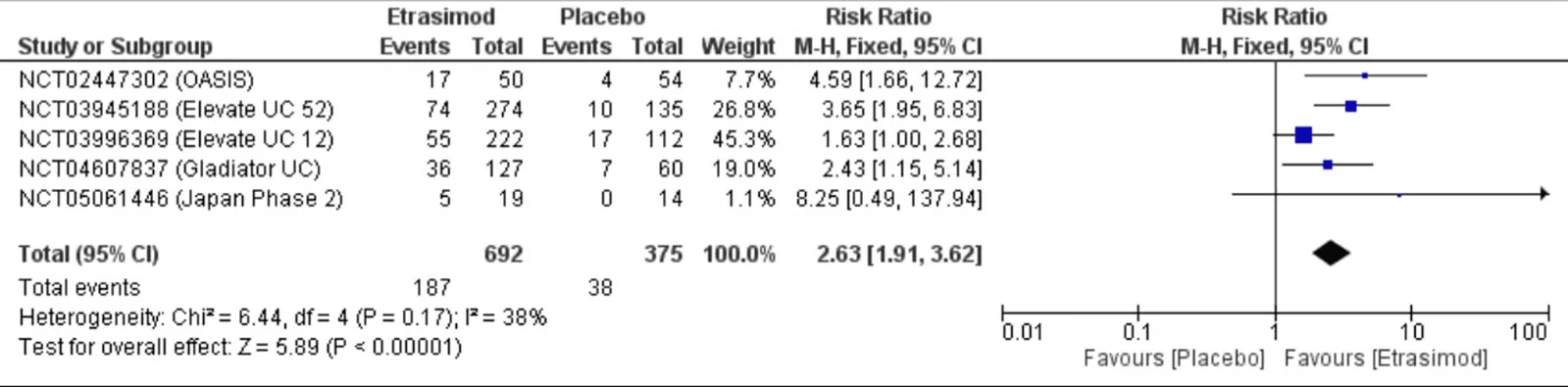
Forest plot demonstrating the effect of Etrasimod on clinical remission at 12 weeks References: [14,16-19]

Clinical response: A statistically significant difference was observed in patients receiving Etrasimod compared with placebo in achieving clinical response at Week 12 (RR 1.68; 95% CI: 1.44, 1.95; p-value < 0.00001). Patients receiving Etrasimod were 1.68 times as likely to achieve clinical response at Week 12 compared with those receiving placebo. No heterogeneity was seen (I² = 0%). Figure 4 illustrates the forest plot of these findings. 

**Figure 4:**
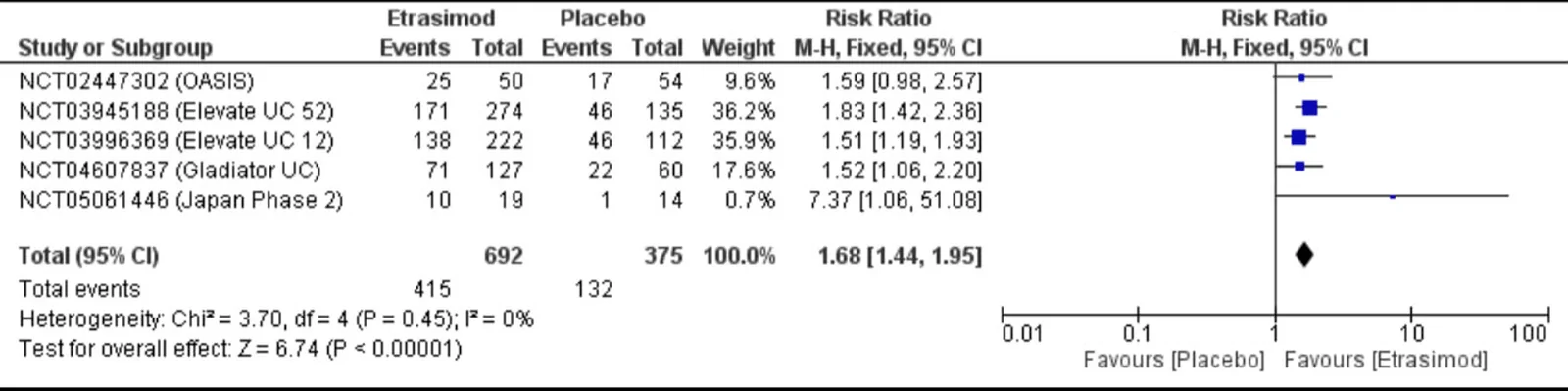
Forest plot demonstrating the effect of Etrasimod on clinical response at 12 weeks References: [14,16-19]

Endoscopic improvement: A statistically significant difference was observed in patients receiving Etrasimod compared with placebo in achieving endoscopic improvement at Week 12 (RR 2.15; 95% CI: 1.67, 2.75; p-value < 0.00001). Patients receiving Etrasimod were 2.15 times as likely to achieve endoscopic improvement at Week 12 compared with those receiving placebo. No heterogeneity was seen (I² = 0%). Figure 5 illustrates the forest plot of these findings. 

**Figure 5:**
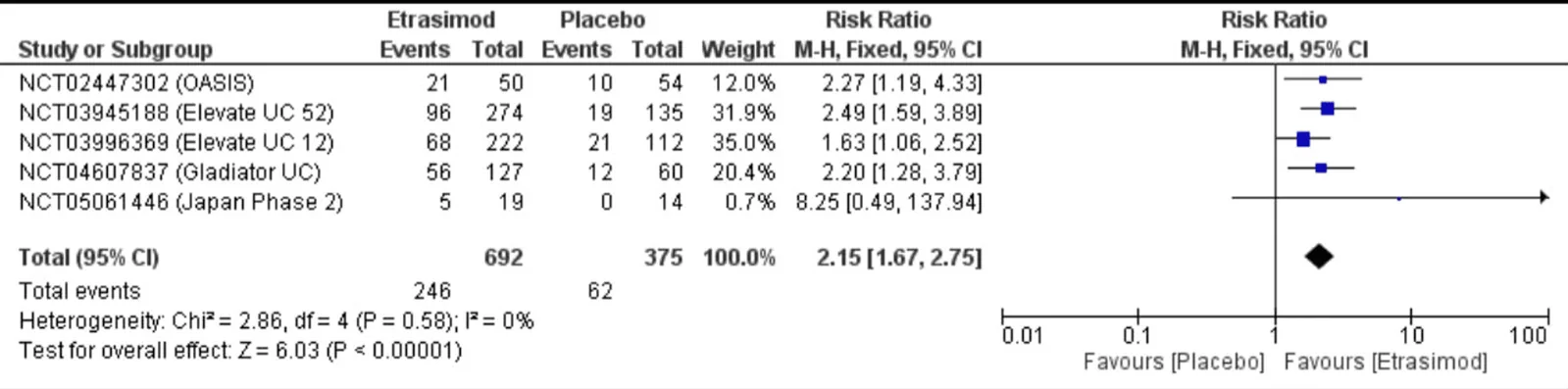
Forest plot demonstrating the effect of Etrasimod on endoscopic improvement at 12 weeks References: [14,16-19]

Symptomatic remission: A statistically significant difference was observed in patients receiving Etrasimod compared with placebo in achieving symptomatic remission at Week 12 (RR 1.81; 95% CI: 1.47, 2.24; p-value < 0.00001). Patients receiving Etrasimod were 1.81 times as likely to achieve symptomatic remission at Week 12 compared with those receiving placebo. The level of heterogeneity was low (I² = 19%). Figure 6 illustrates the forest plot of these findings.

**Figure 6:**
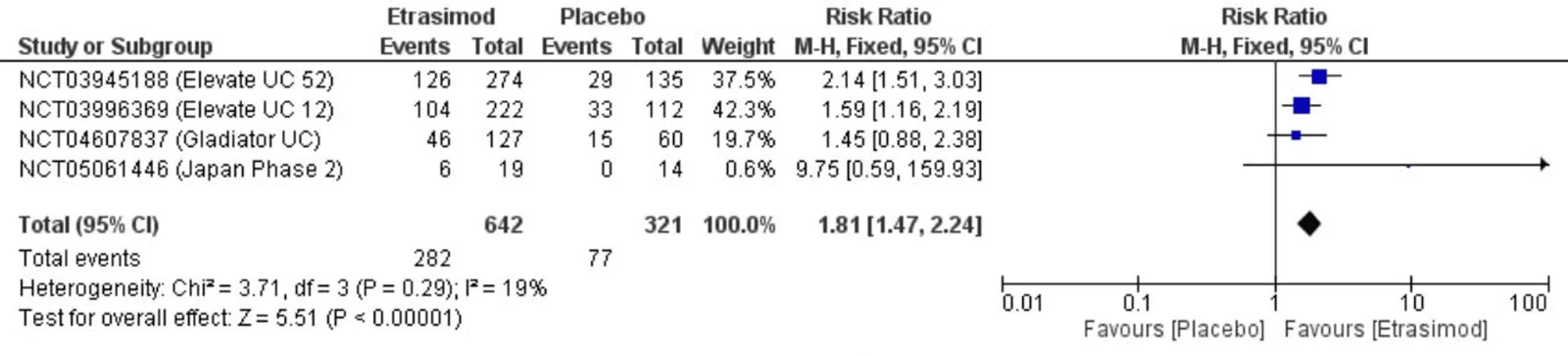
Forest plot demonstrating the effect of Etrasimod on symptomatic remission at 12 weeks References: [16-19]

Mucosal healing: A statistically significant difference was observed in patients receiving Etrasimod compared with placebo in achieving mucosal healing at Week 12 (RR 2.67; 95% CI: 1.77, 4.02; p-value < 0.00001). Patients receiving Etrasimod were 2.67 times as likely to achieve mucosal healing at Week 12 compared with those receiving placebo. The level of heterogeneity was low (I² = 16%). Figure 7 illustrates the forest plot of these findings.

**Figure 7:**
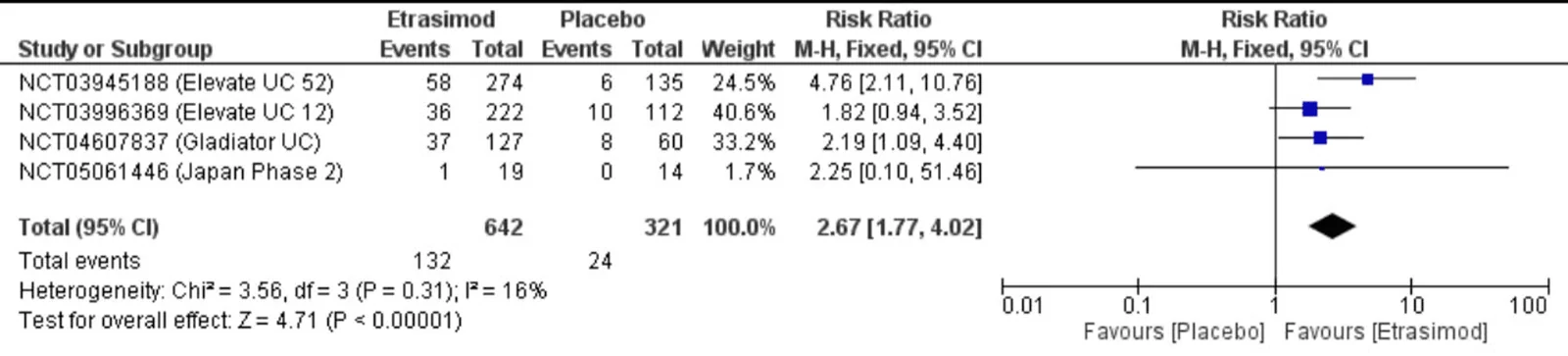
Forest plot demonstrating the effect of Etrasimod on mucosal healing at 12 weeks References: [16-19]

During the maintenance phase, the efficacy of Etrasimod was assessed at >40 weeks across the included studies.

Clinical remission: A statistically significant difference was observed in patients receiving Etrasimod compared with placebo in achieving clinical remission at ≥40 weeks (RR 2.87; 95% CI: 1.89, 4.37; p-value < 0.00001). Patients receiving Etrasimod were 2.87 times as likely to maintain clinical remission at ≥40 weeks compared with those receiving placebo. The level of heterogeneity seen was high (I² = 63%). Figure 8 illustrates the forest plot of these findings. 

**Figure 8:**
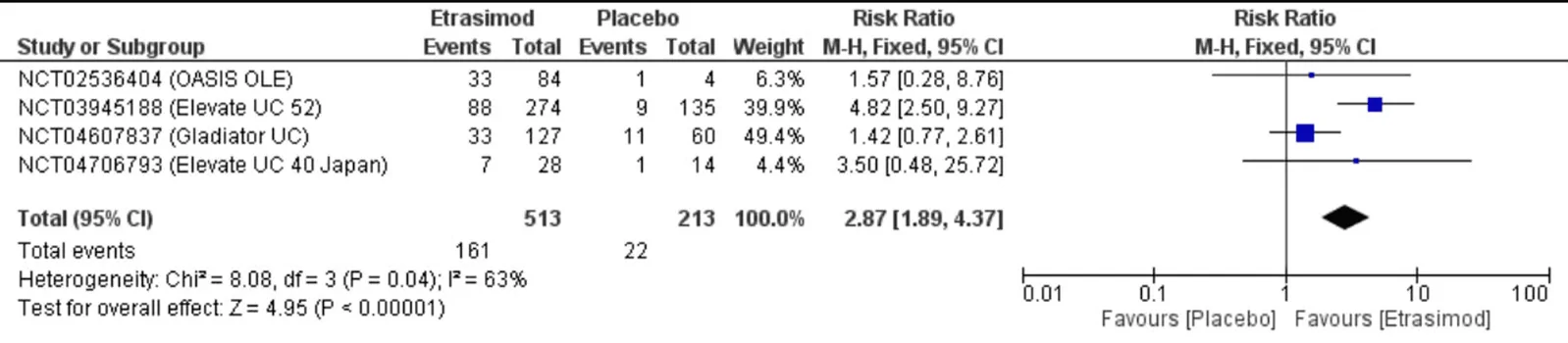
Forest plot demonstrating the effect of Etrasimod on clinical remission at >40 weeks References: [15,16,18,20]

Clinical response: A statistically significant difference was observed in patients receiving Etrasimod compared with placebo in achieving clinical response at ≥40 weeks (RR 1.73; 95% CI: 1.35, 2.21; p-value < 0.0001). Patients receiving Etrasimod were 1.73 times as likely to maintain clinical response at ≥40 weeks compared with those receiving placebo. A high level of heterogeneity was observed (I² = 68%). Figure 9 illustrates the forest plot of these findings.

**Figure 9:**
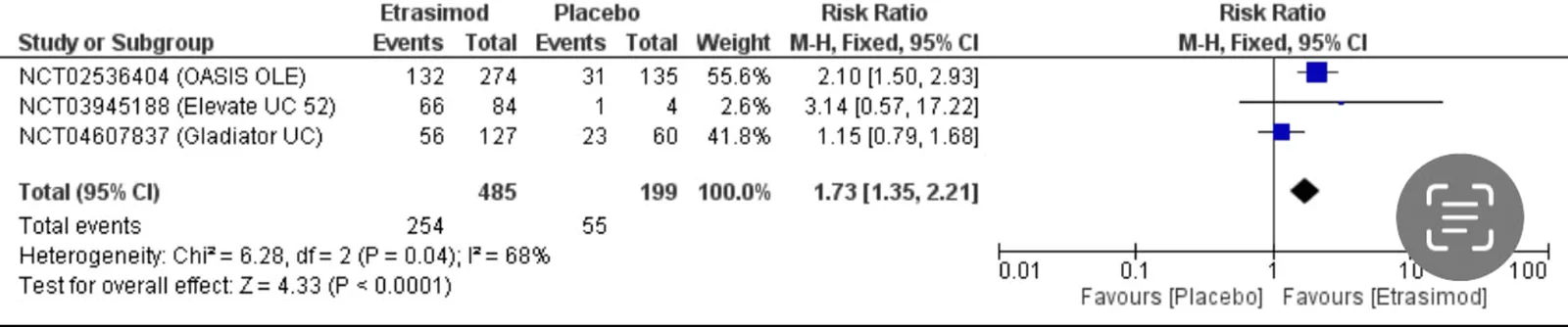
Forest plot demonstrating the effect of Etrasimod on clinical response at >40 weeks References: [15,16,18]

Endoscopic improvement: A statistically significant difference was observed in patients receiving Etrasimod compared with placebo in achieving endoscopic improvement at ≥40 weeks (RR 2.57; 95% CI: 1.79, 3.68; p-value < 0.00001). Patients receiving Etrasimod were 2.57 times as likely to maintain endoscopic improvement at ≥40 weeks compared with those receiving placebo. The level of heterogeneity seen was high (I² = 73%), suggesting variability in long-term follow-up across studies. Figure 10 illustrates the forest plot of these findings.

**Figure 10:**
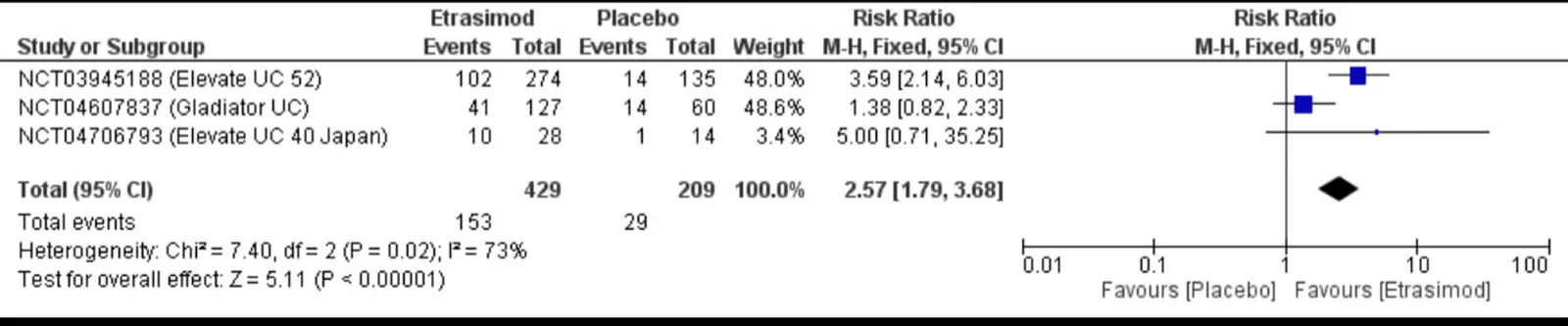
Forest plot demonstrating the effect of Etrasimod on endoscopic improvement at >40 weeks References: [16,18,20]

Symptomatic remission: A statistically significant difference was observed in patients receiving Etrasimod compared with placebo in maintaining symptomatic remission at ≥40 weeks (RR 1.96; 95% CI: 1.47, 2.61; p-value < 0.00001). Patients receiving Etrasimod were 1.96 times as likely to maintain symptomatic remission at ≥40 weeks compared with those receiving placebo. A high level of heterogeneity was observed (I² = 67%). Figure 11 illustrates the forest plot of these findings. 

**Figure 11:**
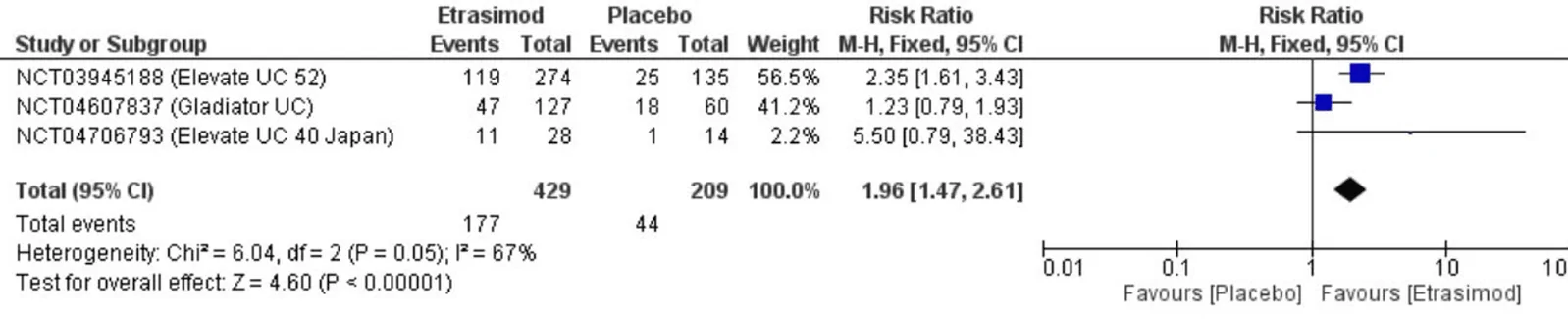
Forest plot demonstrating the effect of Etrasimod on symptomatic remission at >40 weeks References: [16,18,20]

Mucosal healing: A statistically significant difference was observed in patients receiving Etrasimod compared with placebo in maintaining mucosal healing at ≥40 weeks (RR 2.62; 95% CI: 1.69, 4.04; p-value < 0.00001). Patients receiving Etrasimod were 2.62 times as likely to maintain mucosal healing at ≥40 weeks compared with those receiving placebo. Heterogeneity observed was low (I² = 16%). Figure 12 illustrates the forest plot of these findings.

**Figure 12:**
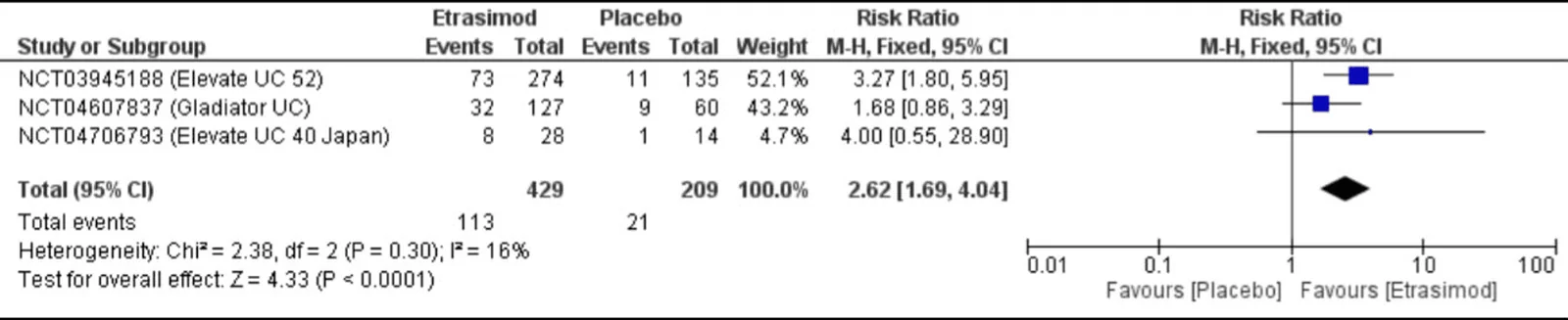
Forest plot demonstrating the effect of Etrasimod on mucosal healing at >40 weeks References: [16,18,20]

Steroid-Free Clinical Remission

A statistically significant difference was observed in patients receiving Etrasimod compared with placebo in achieving steroid-free clinical remission (RR 3.83; 95% CI: 2.21, 6.62; p-value < 0.00001). Patients receiving Etrasimod were 3.83 times as likely to achieve steroid-free clinical remission compared with those receiving placebo. The level of heterogeneity observed was high (I² = 60%). Figure 13 illustrates the forest plot of these findings.

**Figure 13:**
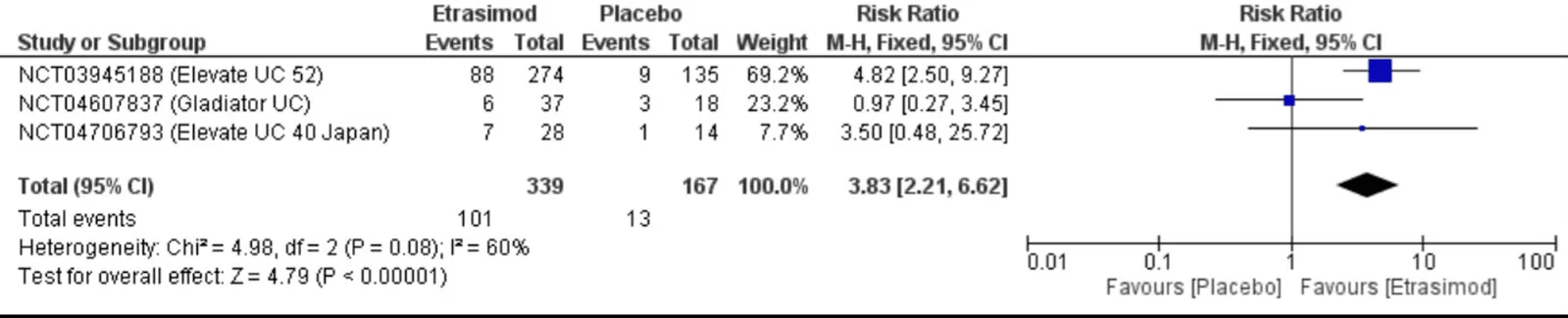
Forest plot demonstrating the effect of Etrasimod on steroid-free clinical remission References: [16,18,20]

Safety Outcomes

Safety outcomes were evaluated by comparing the incidence of serious and treatment-emergent adverse events (TEAEs) between the Etrasimod and placebo groups across all included studies.

Serious Adverse Events

No statistically significant difference was observed between patients receiving Etrasimod and placebo in the incidence of serious adverse events (RR 1.06; 95% CI: 0.61, 1.85; p-value = 0.83). Heterogeneity was low (I² = 14%). Figure 14 illustrates the forest plot of these findings.

**Figure 14:**
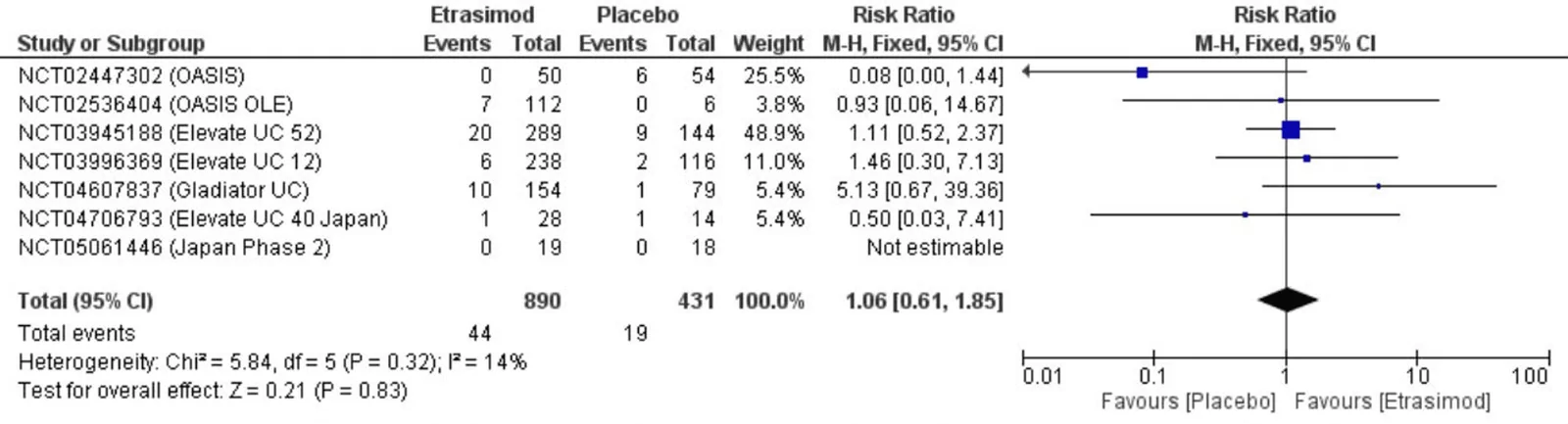
Forest plot demonstrating severe adverse events in patients receiving Etrasimod References: [14-20]

Treatment-Emergent Adverse Events

A statistically significant difference was observed in the incidence of TEAEs between patients receiving Etrasimod and placebo (RR 1.15; 95% CI: 1.03, 1.28; p-value = 0.01). Patients receiving Etrasimod were 1.15 times as likely to experience other adverse events compared with those receiving placebo; however, the increase in risk was small. Moderate heterogeneity was observed (I² = 41%). Figure 15 illustrates the forest plot of these findings.

**Figure 15:**
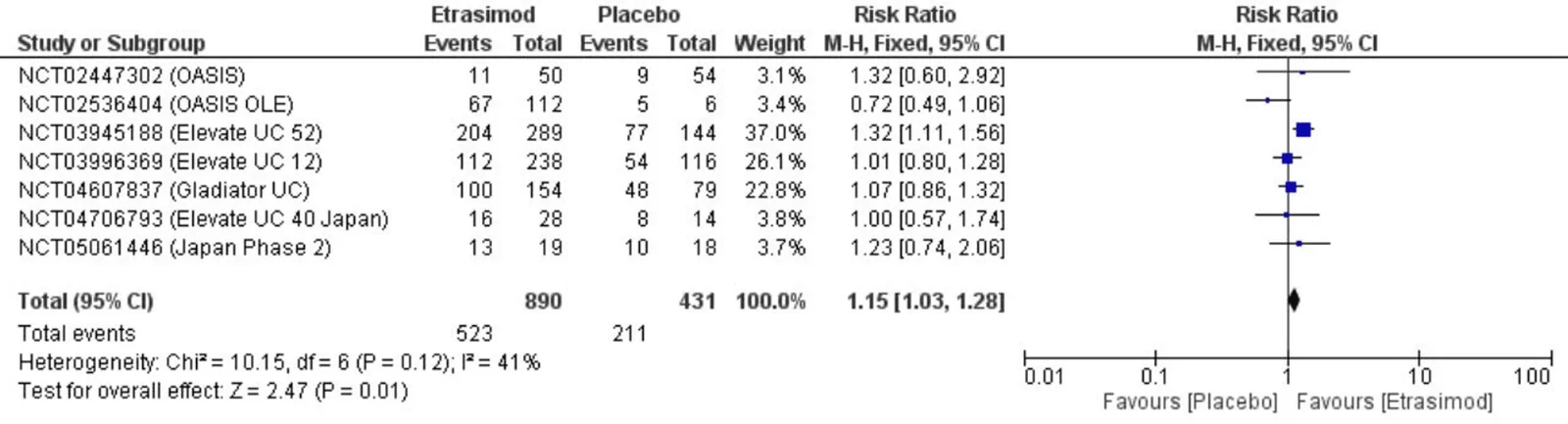
Forest plot demonstrating minor adverse events in patients receiving Etrasimod References: [14-20]

All-Cause Mortality

No deaths were reported in either the Etrasimod or placebo groups across any of the included studies. Hence, a pooled effect estimate could not be calculated. Figure 16 illustrates these findings.

**Figure 16:**
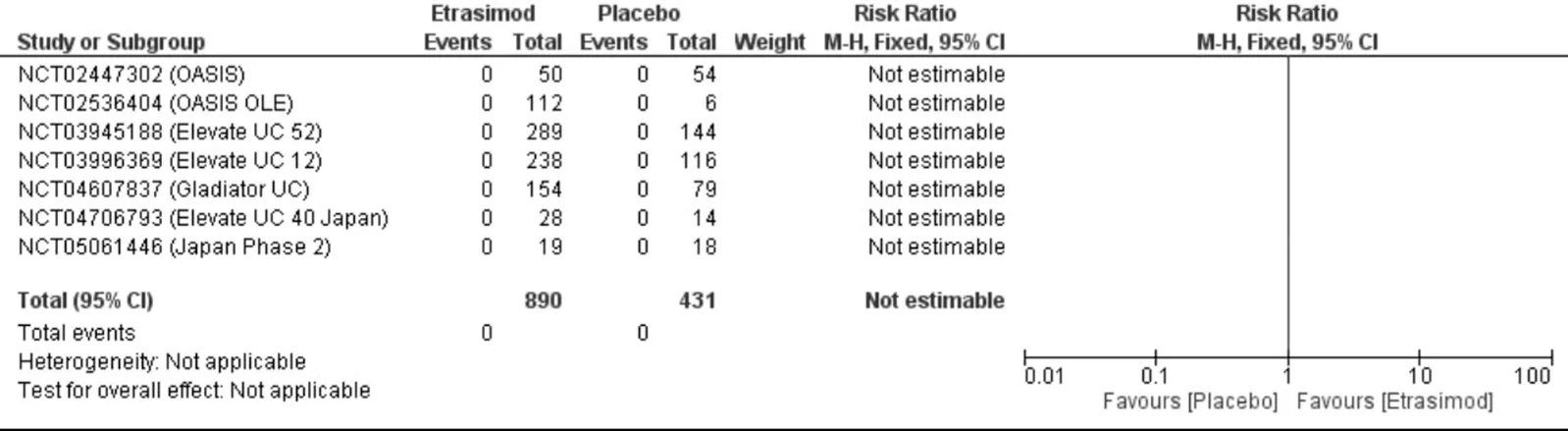
Forest plot demonstrating all-cause mortality in patients receiving Etrasimod References: [14-20]

Discussion

This meta-analysis demonstrated that Etrasimod is associated with significantly improved clinical outcomes in patients with moderate to severe UC. During both the induction and maintenance phases, Etrasimod significantly increased the likelihood of achieving clinical remission, clinical response, endoscopic improvement, symptomatic remission, mucosal healing and steroid-free clinical remission. The treatment effects were generally consistent across studies, although a high level of heterogeneity was observed for maintenance outcomes, probably due to differences in follow-up period ranging from 40 to 52 weeks across studies. Another minor factor that may also have contributed to heterogeneity is the difference between global and Japanese study populations. In terms of safety, the risk of serious adverse events was similar between the Etrasimod and placebo groups, while a very small increase in treatment-emergent adverse events was noted among patients receiving Etrasimod. Common TEAEs reported with Etrasimod include headache, nasopharyngitis, nausea, upper respiratory tract infections and increased alanine aminotransferase (ALT) levels [11]. No deaths were reported in either group across the included studies.

Etrasimod is an S1P receptor modulator that has been approved for the treatment of moderate to severe UC in adults. It selectively targets S1P receptors 1, 4, and 5, which reduces the movement of lymphocytes from lymph nodes into the bloodstream, which ultimately limits their migration to sites of intestinal inflammation. This helps reduce inflammation and improve clinical outcomes in patients with UC. Etrasimod is an orally administered drug that has to be taken once daily, providing a convenient treatment option for patients with active UC in comparison to other advanced therapies, which mostly require injection or infusion. Similar to the findings of our meta-analysis, Glassner et al. also reported that recent trials have demonstrated the potential of Etrasimod to achieve and maintain clinical and endoscopic improvements, supporting its role as a therapeutic option for patients with active UC [21].

The safety findings of our meta-analysis are consistent with the established safety profile of Etrasimod. Similar to our findings, a long-term safety analysis of 956 patients reported serious adverse events in 4.6% of patients receiving Etrasimod compared with 5.4% receiving placebo, with no major new safety concerns after up to 2.5 years of exposure [22]. Lymphopenia, infections such as herpes zoster, cardiovascular effects including bradycardia or conduction abnormalities, macular oedema and hepatic complications remain important safety considerations and highlight the need for appropriate patient selection and regular monitoring during treatment [23]. Overall, the absence of a significant increase in serious adverse events and fatalities in our meta-analysis provides reassurance regarding the short- to medium-duration safety of Etrasimod in patients with moderate to severe disease activity [22,23]. 

A similar meta-analysis published in 2024 reviewed three RCTs to report the efficacy and safety profile of Etrasimod. However, this study was limited by a smaller sample size compared to our updated meta-analysis, which includes five independent RCTs, as well as both short-term and long-term follow-up periods. We divided the analysis into induction at 12 weeks and maintenance outcomes beyond 40 weeks of drug initiation. Our meta-analysis builds on previous research by including more recent RCTs and analysing maintenance outcomes at follow-up periods exceeding 40 weeks. This enhances the strength of our study and provides a more recent picture of the efficacy and safety of Etrasimod [24].

Another more recent meta-analysis by Alla et al. also demonstrated that Etrasimod significantly improves important clinical and endoscopic outcomes in patients with moderate to severe UC. Their analysis included only two studies comprising three RCTs reporting significant improvements in clinical remission, clinical response, and endoscopic improvement. However, our updated meta-analysis provides a more comprehensive assessment by incorporating additional efficacy outcomes, as well as evaluating outcomes during both the induction and maintenance phases. In our meta-analysis, Etrasimod consistently improved these outcomes at both 12 weeks and beyond 40 weeks, suggesting that its benefits are sustained with longer treatment. The safety profile findings reported by this meta-analysis are similar to our results. Overall, the findings of our updated meta-analysis reinforce the previously reported efficacy and favourable safety profile of Etrasimod while providing broader evidence regarding its sustained efficacy and safety across a greater range of clinically relevant outcomes [25].

The RCTs included in our study underwent quality assessment and evaluation for potential risk of bias, demonstrating overall high quality. Data extraction was independently performed by two reviewers to reduce the potential for bias. The data analysis was also carried out by two reviewers independently to improve accuracy and ensure consistent and reliable results. The inclusion of seven reports from five independent RCTs conducted across over 40 countries provides a broader evidence base for assessing the effectiveness and safety profile of Etrasimod.

Limitations of this meta-analysis include using aggregated data from studies, which limits the ability to appreciate individual patient-specific demographic and clinical factors. The included trials also had differences in follow-up periods for maintenance, which may have contributed to some variability and high heterogeneity in some outcomes. The number of available RCTs remains limited, and publication bias cannot be completely ruled out. Selection bias may also be a concern as only RCTs with results published in peer-reviewed journals or those reported on ClinicalTrials.gov were included in our meta-analysis.

Large-scale trials with longer follow-up periods and larger sample sizes covering wider populations are needed to further assess the effectiveness and potential side effects of Etrasimod. Further studies are required to understand the effects of Etrasimod in patients with varying clinical characteristics and comorbidities. Future studies involving diverse patient populations would help establish the effectiveness of Etrasimod across different clinical settings.

## Conclusions

This updated meta-analysis demonstrates that Etrasimod is an effective treatment for achieving and maintaining remission in patients with moderate to severely active UC. Etrasimod significantly improved clinical outcomes during both induction at 12 weeks and maintenance beyond 40 weeks. The safety findings also support a favourable safety profile of Etrasimod. Overall, these findings support Etrasimod as an effective and well-tolerated treatment option in moderate to severe UC. Further research and trials with larger sample sizes, longer follow-up and more diverse patient populations are needed to confirm the long-term efficacy and safety of Etrasimod and to better understand its effectiveness across different clinical settings.
